# PubChemRDF: towards the semantic annotation of PubChem compound and substance databases

**DOI:** 10.1186/s13321-015-0084-4

**Published:** 2015-07-14

**Authors:** Gang Fu, Colin Batchelor, Michel Dumontier, Janna Hastings, Egon Willighagen, Evan Bolton

**Affiliations:** National Center for Biotechnology Information, National Library of Medicine, National Institute of Health, Bethesda, MD USA; Royal Society of Chemistry, Thomas Graham House, Cambridge, UK; Stanford Center for Biomedical Informatics Research, Stanford University, Stanford, USA; European Molecular Biology Laboratory-European Bioinformatics Institute (EMBL-EBI), Hinxton, UK; Department of Bioinformatics-BiGCaT, NUTRIM, Maastricht University, Maastricht, The Netherlands

## Abstract

**Background:**

PubChem is an open repository for chemical structures, biological activities and biomedical annotations. Semantic Web technologies are emerging as an increasingly important approach to distribute and integrate scientific data. Exposing PubChem data to Semantic Web services may help enable automated data integration and management, as well as facilitate interoperable web applications.

**Description:**

This work, one of a series covering the PubChemRDF project, describes an approach to translate PubChem Substance and Compound information into Resource Description Framework (RDF) format. Basic examples are provided to demonstrate its use. The aim of this effort is to provide two new primary benefits to researchers in a cost-effective manner. Firstly, we aim to remove the inherent limitations of using the web-based resource PubChem by allowing a researcher to use readily available semantic technologies (namely, RDF triple stores and their corresponding SPARQL query engines) to query and analyze PubChem data on local computing resources. Secondly, this work intends to help improve data sharing, analysis, and integration of PubChem data to resources external to NCBI and across scientific domains, by means of the association of PubChem data to existing ontological frameworks, including CHEMical INFormation ontology, Semanticscience Integrated Ontology, and others.

**Conclusions:**

With the goal of semantically describing information available in the PubChem archive, pre-existing ontological frameworks were used, rather than creating new ones. Semantic relationships between compounds and substances, chemical descriptors associated with compounds and substances, interrelationships between chemicals, as well as provenance and attribute metadata of substances are described.

**Electronic supplementary material:**

The online version of this article (doi:10.1186/s13321-015-0084-4) contains supplementary material, which is available to authorized users.

**Graphical abstract:**
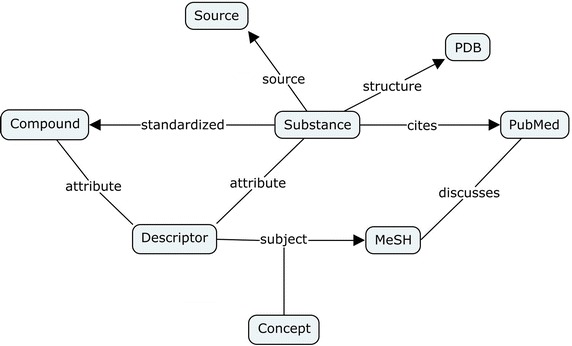
Schematic representation of the semantic links for PubChem compounds and substances.

## Background

PubChem [1, 2] is an open repository for chemical substance description, biological activities and biomedical annotations. PubChem is organized as three distinct and interrelated primary databases: Substance, BioAssay, and Compound. The Substance database (accession SID) includes depositor-provided sample description information including chemical depictions, chemical names (synonyms), external registration identifiers, comments, and cross-links. The BioAssay database (accession AID) includes depositor-provided experimental result information, including experiment description, experiment protocol, and results for substance SIDs tested in a biological assay. The nature of the assay tests can be rather diverse, including phenotypic, against a defined target, high-throughput, dose–response, counter-screen, or physical property measurement. Contributors providing cross-links with their data help to integrate and cross-reference PubChem to other National Center for Biotechnology Information (NCBI) resources (like PubMed) and beyond (for example, other chemical biology resources and patent documents).

PubChem, as an archive, takes care to preserve the provenance of information. Each change to a contributed substance or assay made by the depositor is versioned. In addition, each PubChem depositor controls their own records. As such, there can be many providers of information about a particular chemical substance (e.g., aspirin).

One of the key purposes of the Compound database is to help aggregate information from various PubChem contributors using the chemical structure as the key [2]. The Compound database (accession CID) contains the unique chemical structure abstracted from the Substance database. As a part of this, each substance record with chemical information is subjected to a validation and normalization procedure to ensure the chemical structure is well-defined (e.g., no variable or ill-defined information), makes chemical sense (e.g., it is very unlikely that five bonds are connected to a carbon atom in small molecules of pharmacological interest), and to provide a standard chemical representation (e.g., collapse functional group and tautomeric/resonance variation into an equivalent, single, canonic form, as-is possible). Structures stored within PubChem Compound are cross-referenced and pre-clustered by identity and similarity group concepts [2]. In addition, PubChem Compound employs chemical name/structure consistency analysis [3]. This processing, and the aforementioned structure normalization, helps to reduce error proliferation by informing contributors of any potential issues found with in-coming data prior to it being loaded into PubChem, and by suppressing potentially anomalous depositor-provided information by default in PubChem Compound interfaces. In addition, for each Compound record, PubChem calculates 3-D coordinates [4], physical properties (e.g., molecular weight, XLogP3 [5]), and descriptors (e.g., InChI [6], SMILES [7, 8], IUPAC names [9]), as-is possible.

In order to facilitate data integration of public chemical information, more and more efforts have taken advantage of the common data format and representation backed by the controlled vocabularies with well-defined semantics [10, 11]. Linked Data [12] built for the Semantic Web (as a collection of technologies and standards) [13] offers an approach to share data using web technologies. Semantic Web technologies offer a well-defined syntax and semantics for the formal representation of and reasoning with domain knowledge. The formalization of PubChem knowledge provides clarity by defining the meaning of entity attributes and relations in a machine interpretable manner. Moreover, harnessing ontologies for knowledge description can promote the interoperability of PubChem data with other domain knowledge including systems biology [14], and translational medicine research [15], among others. The semantic annotation of PubChem databases can directly promote the interoperability between applications external to NCBI Entrez and PubChem interfaces.

Of key importance, Semantic Web technologies and standards include the trio of the Resource Description Framework (RDF), Web Ontology Language (OWL), and SPARQL query language [16]. RDF is a standard model that uses machine-understandable metadata to describe the type and relation of any Web resource, which can be anything that has an identity, such as a document, a person, a datum, or an operation. RDF uses an abstract model to decompose information into small pieces with well-defined semantics (meaning), so as to express knowledge in a general, yet simple and flexible way. Each small piece of information is represented as an RDF statement, also called a “triple” of subject-predicate-object, and the RDF model can be expressed as a collection of triples. The semantics and syntax in a given RDF model are defined in controlled vocabularies or ontologies, and OWL is widely used to create domain-specific ontologies with increased expressivity. It is worth noting that ontologies are not only vocabularies that define a set of common and shared terms in a hierarchical structure to describe domain knowledge, they are also computable by enabling first-order logical reasoning, i.e. the statements asserted to the parent classes can be inherited by the child classes. The logic-based inference can be used to derive new RDF statements that are not explicitly asserted, and logic rules can be used to identify conflict statements on behalf of consistency checking. Hence, ontologies designed for automated inference must be carefully formulated according to the semantics of the language and as such are distinct from informal knowledge organization systems such as taxonomy and thesaurus. SPARQL serves as an RDF query language and data access protocol for the Semantic Web with the ability to locate and retrieve specific information across widespread databases as well as generate query reports that can be directly analyzed by network visualization and data mining applications. SPARQL may be used to query relational databases [17, 18] as well as RDF databases (triple stores) [19, 20], and may increase in popularity in the near future with the rapidly increasing scalability of RDF databases. With all of this in mind, PubChem data described using existing ontology frameworks and published in RDF format could fulfill the equivalent need for a SQL-based database of PubChem. Once PubChem data in RDF format is loaded into an RDF triple store, it should be immediately usable to researchers for complex queries and data analysis in their own local compute environment, whether it is a desktop computer or a multi-server compute farm. The compressed RDF-formatted PubChem data is more compact than the equivalent PubChem SQL-based databases, helping to make data distribution more tractable. With RDF-formatted data, a documented SQL schema of PubChem data is no longer required, as the ontology linked to the data provides the necessary documentation. As long as the PubChemRDF data mapping is stable, development changes to PubChem internal specialized systems can happen without impacting the PubChemRDF data. Therefore, in theory, Semantic Web technologies could provide the basis to replace the need for a SQL-based PubChem data system.

Providing scientific data similar to that contained within PubChem in RDF format is not without precedent. Other RDF-based resources exist, such as European Bioinformatics Institute (EBI) RDF [21], Bio2RDF [22, 23], Linked Open Drug Data (LODD) [24], Chem2Bio2RDF [25], Open PHACTS [26], ChEMBL RDF [27], and others. The EBI RDF platform encompasses six public life science databases including ChEMBL, UniProt, Reactome, BioModels, BioSamples, and Expression Atlas. Bio2RDF serves as a mash-up system that integrates publicly available bioinformatics databases to provide interlinked life science data (~4 billion RDF triples). The LODD project, led by the World Wide Web Consortium (W3C) Health Care and Life Science Interest Group (HCLS IG), interlinks twelve open-access drug databases related to pharmaceutical research and development within the linked data cloud (~8 million triples). Chem2Bio2RDF is designed for integrated network analysis of heterogeneous datasets across the chemical and biological domains. It provides a computational tool for systems chemical biology and chemogenomics studies by aggregating multiple repositories cross-linked between Bio2RDF and LODD. Open PHACTS (Pharmacological Concept Triple Store) under the European Innovative Medicines Initiative (IMI) develops new solutions to create a public, integrated and sustainable Open Pharmacological Space (OPS) platform serving as an open source, open standard, open access infrastructure for drug discovery research. For example, standards are proposed on how to describe data sets and how to semantically link chemical compounds between databases [28].

With these precedents in mind, this work, one of a series covering the PubChemRDF project, describes how we translate PubChem substance and compound data into RDF format. Basic examples are provided to demonstrate its use. The aim of this effort is to provide two new primary benefits to researchers in a cost-effective manner. Firstly, the PubChemRDF project intends to remove the inherent limitations of using the web-based PubChem resource (such as limitations on query frequency or the inability to construct complicated queries using the available web-based interfaces) by allowing a researcher to use readily available semantic technologies (namely, RDF triple stores and their corresponding SPARQL query engines) to query and analyze PubChem data on local computing resources. Secondly, the PubChemRDF project intends to help improve data sharing, analysis, and integration of PubChem data to resources external to NCBI and across scientific domains by means of the association of PubChem data to existing ontological frameworks.

## Construction and content

The PubChemRDF content covered in the scope of this paper includes the core chemical information archived in the PubChem Compound and Substance databases, the semantic relationships between compounds and substances, the chemical descriptors associated with compounds and substances, the interrelationships between compounds, and the provenance and attribution metadata of substances. The corresponding RDF statements to describe these will be demonstrated in the following sections. A set of standardized ontologies for enhanced data integration and interoperability were collected to define the domain-specific knowledge, including Chemical Entities of Biological Interest (ChEBI) [29–31], CHEMical INFormation ontology (CHEMINF) [32], Semanticscience Integrated Ontology (SIO) [33], Units of Measurement (UO) [34], Dublin Core Metadata Initiative (DCMI) Terms [35], Citation Typing Ontology (CiTO) [36], and Simple Knowledge Organization System (SKOS) [37]. The ontologies ChEBI, CHEMINF, SIO, and UO are interfaced by the NIH Roadmap National Center for Biomedical Ontology (NCBO) through its BioPortal [38], and comply with an evolving set of shared principles established by the Open Biomedical Ontologies (OBO) foundry [39]. Adoption of these core ontologies helps to ensure that the mapping of chemical information is compatible across multiple Semantic Web resources.

RDF statements described here are written in the Turtle syntax [40] with uniform resource identifier (URI) [41] references in relative form. The Turtle prefix directives for the namespaces of PubChem subdomains and the aforementioned ontologies are listed in Table 1, which can be used to resolve the base URIs relative to the fragment (local) identifiers. Both 303 URI (303 redirection) and hash URI were employed in the PubChemRDF project according to W3C recommendation [42]. Hash URI with a ‘#’ sign between the base URI and the fragment identifier was only used for PubChem vocabulary, which defines the types and relations of some PubChem-specific terms that cannot be identified in standardized ontologies. The 303 URI with a ‘:’ sign between the base URI and the fragment identifier was used for the other PubChemRDF subdomains (see Table 1). The fragment identifiers for PubChem Compound and Substance are constructed based on the CIDs and SIDs, respectively. The URIs for atorvastatin in PubChem Compound database [PubChem: CID60823] and Substance database [PubChem: SID103554720] are assigned as:

$$\begin{aligned} &\tt{http{{:}}//rdf.ncbi.nlm.nih.gov/pubchem/compound/CID60823} \\ &\tt{http{{:}}//rdf.ncbi.nlm.nih.gov/pubchem/substance/SID103554720} \end{aligned}$$

**Table 1:** The prefixes and corresponding namespaces of PubChem subdomains and standardized ontologies

Prefix^a^	Namespace^b^
PubChemRDF subdomains	
compound	http://rdf.ncbi.nlm.nih.gov/pubchem/compound/
substance	http://rdf.ncbi.nlm.nih.gov/pubchem/substance/
descr	http://rdf.ncbi.nlm.nih.gov/pubchem/descriptor/
inchikey	http://rdf.ncbi.nlm.nih.gov/pubchem/inchikey/
syno	http://rdf.ncbi.nlm.nih.gov/pubchem/synonym/
concept	http://rdf.ncbi.nlm.nih.gov/pubchem/concept/
reference	http://rdf.ncbi.nlm.nih.gov/pubchem/reference/
nbr	http://rdf.ncbi.nlm.nih.gov/pubchem/neighbor/
source	http://rdf.ncbi.nlm.nih.gov/pubchem/source/
vocab	http://rdf.ncbi.nlm.nih.gov/pubchem/vocabulary#
External RDF resources	
pdbr	http://rdf.wwpdb.org/pdb/
mesh	http://id.nlm.nih.gov/mesh/
chembl	http://rdf.ebi.ac.uk/resource/chembl/molecule/
linkedchem	http://linkedchemistry.info/chembl/chemblid/

Prefix^a^	Namespace^b^	Vocabularies
Existing ontologies		
rdfs	http://www.w3.org/2000/01/rdf-schema#	RDF schema [55]
rdf	http://www.w3.org/1999/02/22-rdf-syntax-ns#	RDF [56]
xsd	http://www.w3.org/2001/XMLSchema#	XML schema [57]
obo	http://purl.obolibrary.org/obo/	ChEBI [29–31] and UO [34]
sio/cheminf	http://semanticscience.org/resource/ ^b^	CHEMINF [32] and SIO [33]
cito	http://purl.org/spar/cito/	CiTO [36]
pdbo	http://rdf.wwpdb.org/schema/pdbx-v40.owl#	PDB ontology
skos	http://www.w3.org/2004/02/skos/core#	SKOS [37]
dcterms	http://purl.org/dc/terms/	DCMI terms [35]

^a^Prefix substitutes full URI namespace in the context of XML qualified name (QName).

^b^Namespaces can be associated with element and attribute names in URI references; SIO and CHEMINF share the same namespace.

which can be represented in the relative form as $\tt{compound{:}CID60823}$, and $\tt{substance{:}SID103554720}$, respectively.

The fragment identifiers prefixed with the chemical descriptor namespace were constructed based on a combination of primary accession identifiers (CID or SID) and descriptor labels, except the depositor-provided synonyms. For instance, the URI for the molecular weight of CID60823 is represented as:

$$\tt{http{{:}}//rdf.ncbi.nlm.nih.gov/pubchem/descriptor/CID60823\_Molecular\_Weight}$$

which can be abbreviated as $\tt{descr{:}CID60823\_Molecular\_Weight}$. Given the fact that InChIKey is widely used to identify chemical structures and its value has a consistent pattern, which is good for URI construction, a separate namespace for the InChIKey subdomain has been created, which can be used to integrate chemical information from different RDF-based resources. The URI reference for the InChIKey is constructed based on its value as:

$$\tt{http{:}//rdf.ncbi.nlm.nih.gov/pubchem/inchikey/XUKUURHRXDUEBC}{\texttt{-}}\tt{KAYWLYCHSA}{\texttt{-}}\tt{N}$$

which can be abbreviated as $\tt{inchikey{:}XUKUURHRXDUEBC}{\texttt{-}}\tt{KAYWLYCHSA}{\texttt{-}}\tt{N}$. Each SID may be associated with multiple depositor-provided synonyms, and vice versa. For instance, SID103554720 has three depositor-provided synonyms including atorvastatin, ChEMBL identifier and ChEBI identifier; similarly, the chemical name atorvastatin has been associated with more than 70 different substances, including SID103554720, SID210282077, SID210279754, and so on. Moreover, the associations between the substances and synonyms are updated by depositors as a function of time. Hence, in order to disambiguate the URI references for the depositor-provided synonyms, an independent synonym subdomain was adopted to facilitate semantic integration based on chemical names. Since chemical names can be strangely long and may contain URL-encoding reserved characters, the MD5 hash values derived from the depositor-provided synonyms (one-to-one without clash) were used to construct URIs. For instance, the depositor-provided synonym of atorvastatin is represented as:

$$\tt{http{:}//rdf.ncbi.nlm.nih.gov/pubchem/synonym/MD5\_9a05646d461669f86de312d88ab5748a}$$

which can be abbreviated as $\tt{syno{:}MD5\_9a05646d461669f86de312d88ab5748a}$. All of the depositor-provided synonyms were changed to lower case before generating MD5 hash values, so the synonym URIs are dereferenced in a case-insensitive manner.

### PubChem substance

**Figure 1:**
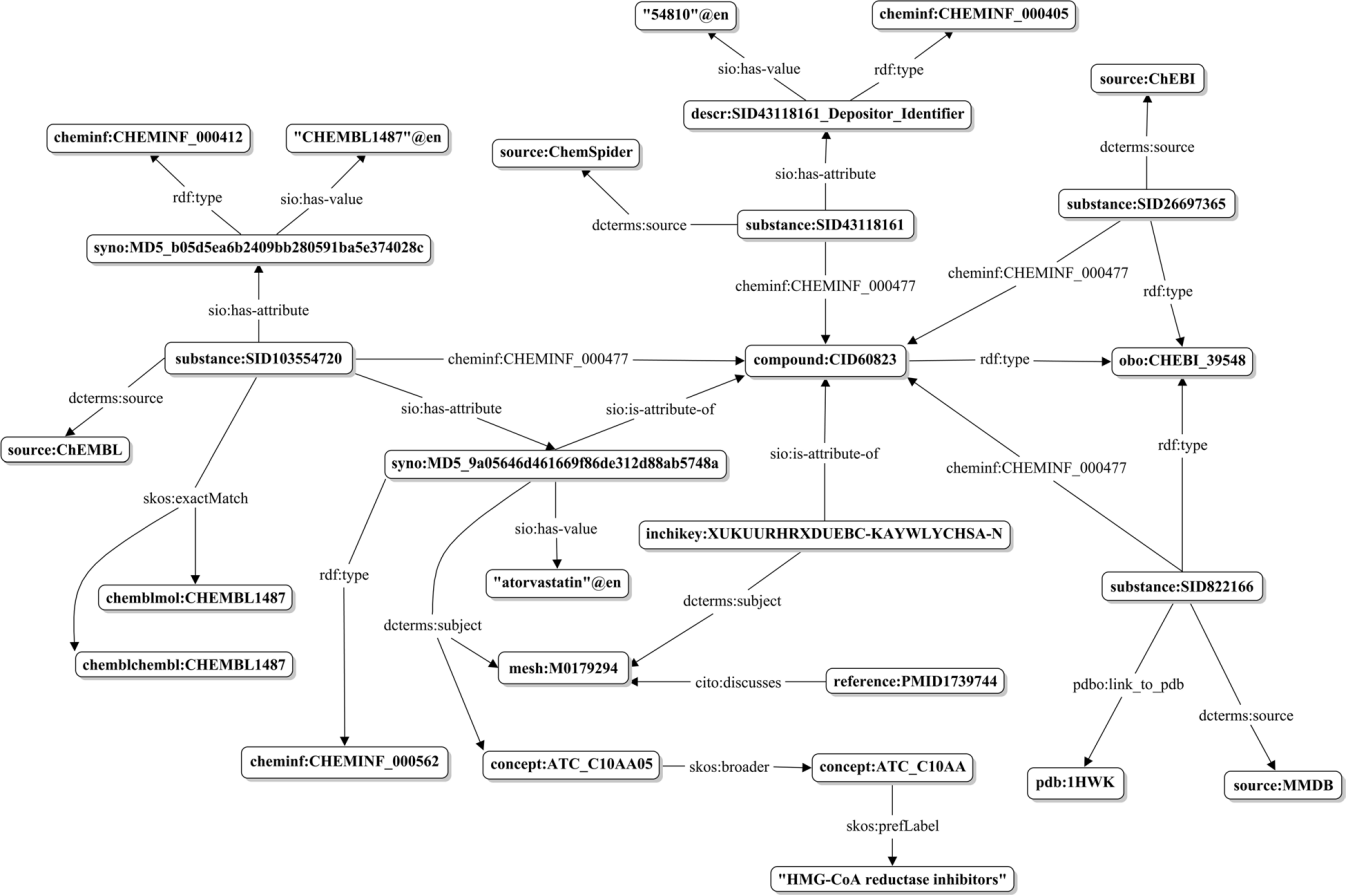
RDF diagram representing the attributes for substances SID103554720, SID43118161, SID26697365, SID822166, and compound CID60823, as well as the annotations for synonym and InChIKey instances.

**Figure 2:**
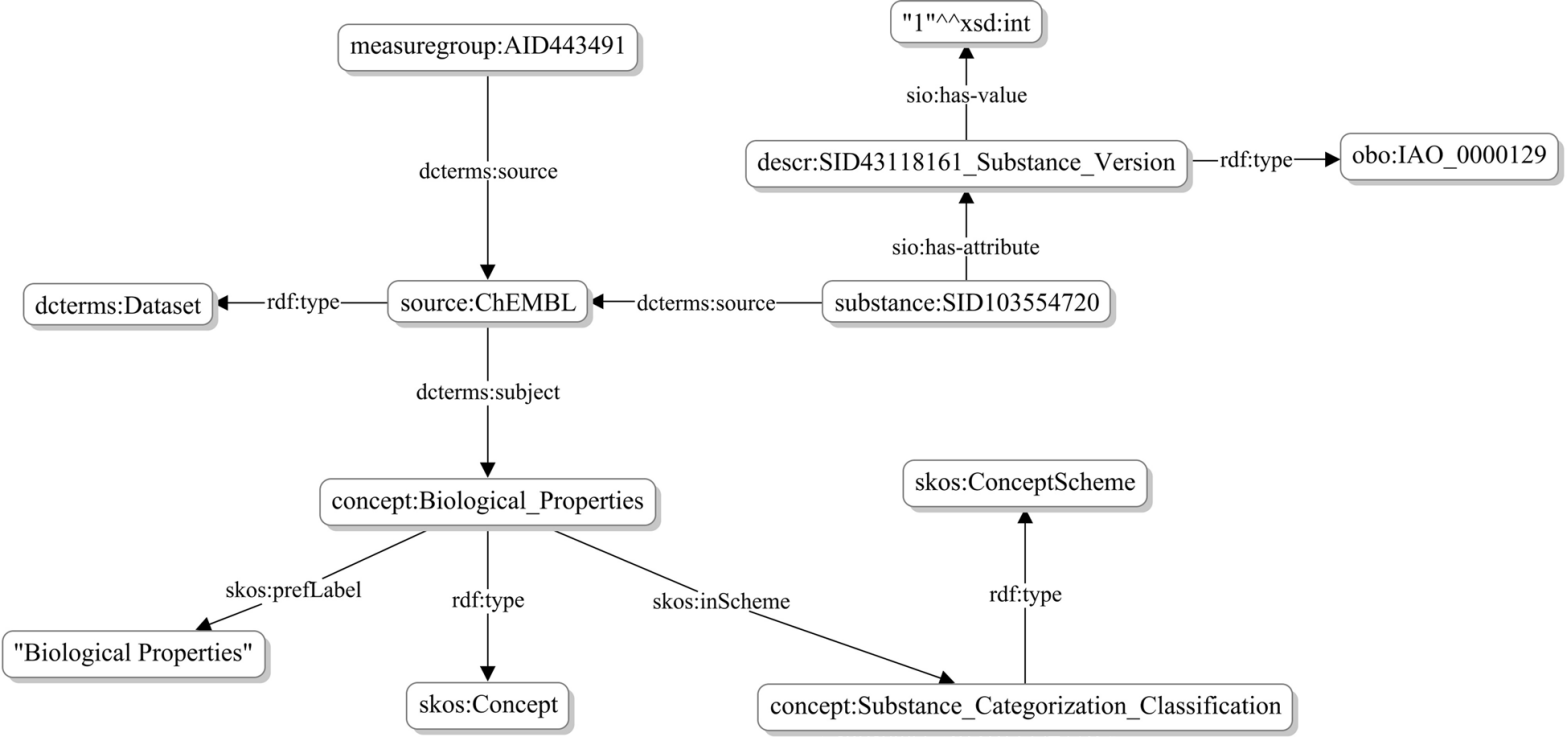
RDF diagram representing PubChem data provenance model.

Every PubChem substance is attributed to one and only one depositor, and the provenance metadata is exposed by using the predicate $\tt{dcterms{:}source}$ (see Figures 1, 2):

$$\tt{substance{:}SID103554720 \,\,dcterms{:}source\,\,source{:}ChEMBL.}$$

The RDF descriptions for ChEMBL are exposed in the data source subdomain. PubChem standardizes chemical structure representations for the purpose of aggregating substance-centric information. This standardization processing includes multiple validation and normalization steps. The validation steps are used to confirm the structural representation is chemically reasonable, being comprised of known atomic elements and with reasonable atomic valence. The normalization steps are used to produce a single chemical structure description from a multitude of effectively equivalent chemical representations (at standard temperature and pressure). Often, the resulting canonical representation is a different tautomeric and resonance form than that originally provided by the PubChem Substance contributor. In most cases, this PubChem derived canonic representation can be considered an equivalent form to that provided by the depositor; however, it is possible that the depositor-provided tautomer is isolatable, more stable, and specifically intended. When standardization processing succeeds, there will be a PubChem Compound record associated to the corresponding substance record. If the standardization processing of a substance fails, for any reason, no compound record will be associated with the given substance record. The association between a PubChem substance and the corresponding PubChem compound is represented by using the predicate $\tt{cheminf{:}CHEMINF\_000477}$ (see Table 2):

$$\tt{substance{:}SID103554720\,\,cheminf{:}CHEMINF\_000477\,\,compound{:}CID60823.}$$

**Table 2:** CHEMINF IDs, corresponding labels, and definitions of terms used to annotate interrelationship between compounds and substances

CHEMINF term ID	Label	Definition
CHEMINF_000477	Has PubChem normalized counterpart	Non-symmetric^a^ predicate between substance as domain^b^ and compound as range^c^
CHEMINF_000480	Has component with uncharged counterpart	Non-symmetric predicate between a mixture compound as domain and its component as range
CHEMINF_000455^d^	Is isotopologue of	Symmetric^e^ predicate between two compounds (isotopomers)
CHEMINF_000461^d^	Is stereoisomer of	Symmetric predicate between two compounds (stereoisomers)
CHEMINF_000462	Has same connectivity as	Symmetric predicate between two compounds with same connectivity
CHEMINF_000482	Similar to by PubChem 2-D similarity algorithm	Symmetric predicate between two similar compounds according to 2-D Tanimoto score
CHEMINF_000483	Similar to by PubChem 3-D similarity algorithm	Symmetric predicate between two similar compound according to 3-D Shape and Color Tanimoto scores

^a^Non-symmetric means the subject and object in the triple are not interchangeable.

^b^Domain is the subject of triple.

^c^Range is the object of triple.

^d^The predicate is sub-property of CHEMINF_000462.

^e^Symmetric means the subject and object in the triple are interchangeable.

PubChem substances are associated with two kinds of attributes: versions and synonyms. The links between the substance and its attribute are exposed as (see Figures 1, 2):

$$\begin{aligned} &\tt{substance{:}SID43118161\,\,sio{:}has}{\texttt{-}}\tt{ attribute} \\ &\quad \tt{descr{:}SID43118161\_Substance\_Version.} \\& \tt{substance{:}SID103554720\,\,sio{:}has}{\texttt{-}}\tt{attribute} \\ &\quad \tt{syno{:}MD5\_b05d5ea6b2409bb280591ba5e374028c.} \end{aligned}$$

The types and values of the versions and synonyms are exposed in the descriptor and synonym subdomains.

If a PubChem Substance was deposited by ChEBI, it is represented as an instance of the corresponding ChEBI ontology class, by using the predicate rdf:type. If this substance has a standardized structure representation in PubChem Compound database, the corresponding compound and all of the other substances standardized to the same compound are exposed as instances of the same ChEBI ontology class. Such knowledge representation situates the PubChem Substance records within the context of the global linked open data project, and enables logic-based inference. For instance, a given ChEBI ontology class [$\tt{obo{:}CHEBI\_39548(atorvastatin)}$] has multiple instances sharing the same canonic structural representation, including $\tt{substance{:}SID26697365, substance{:}SID43118161, substance{:}SID822166, substance{:}SID103554720}$, and $\tt{compound{:}CID60823}$. Based on ChEBI ontological representation, we can infer the fact that all of those instances have pharmacological role: “hydroxymethylglutaryl-CoA (HMG-CoA) reductase inhibitor”. The inferred fact agrees well with the synonym annotation ($\tt{concept{:}ATC\_C10AA}$) defined by the World Health Organization (WHO) anatomical therapeutic chemical (ATC) (see Figure 1).

If a PubChem substance was deposited by Molecular Modeling Database (MMDB) [43], it is most likely co-crystalized with a macromolecule (protein, RNA, or DNA) in an experimental 3-D structure. If the Protein Data Bank (PDB) cross reference for the given MMDB record is provided, a link between the PubChem substance and the PDB record is exposed:

If a PubChem substance was deposited by ChEMBL, it is cross-linked to EBI RDF [21] and ChEMBL RDF [27]:

$$\tt{substance{:}SID822166\,\,pdbo{:}link\_to\_pdb\,\,pdbr{:}1HWK.}$$

$$\begin{aligned}&\tt{substance{:}SID103554720 \,\,skos{:}exactMatch\,\,chembl{:}CHEMBL1487.} \\ &\tt{substance{:}SID103554720\,\,skos{:}exactMatch\,\,linkedchem{:}CHEMBL1487.}\end{aligned}$$

If a PubChem depositor provided the PubMed references for a given substance, the literature mentioning of a given substance is exposed:

$$\tt{substance{:}SID1950 \,\,cito{:}isDiscussedBy\,\, reference{:}PMID11676470.}$$

where the link between a substance and its related reference is provided by the PubChem depositor, so the provenance metadata of the link is same as the provenance metadata of the substance.

### PubChem compound

**Figure 3:**
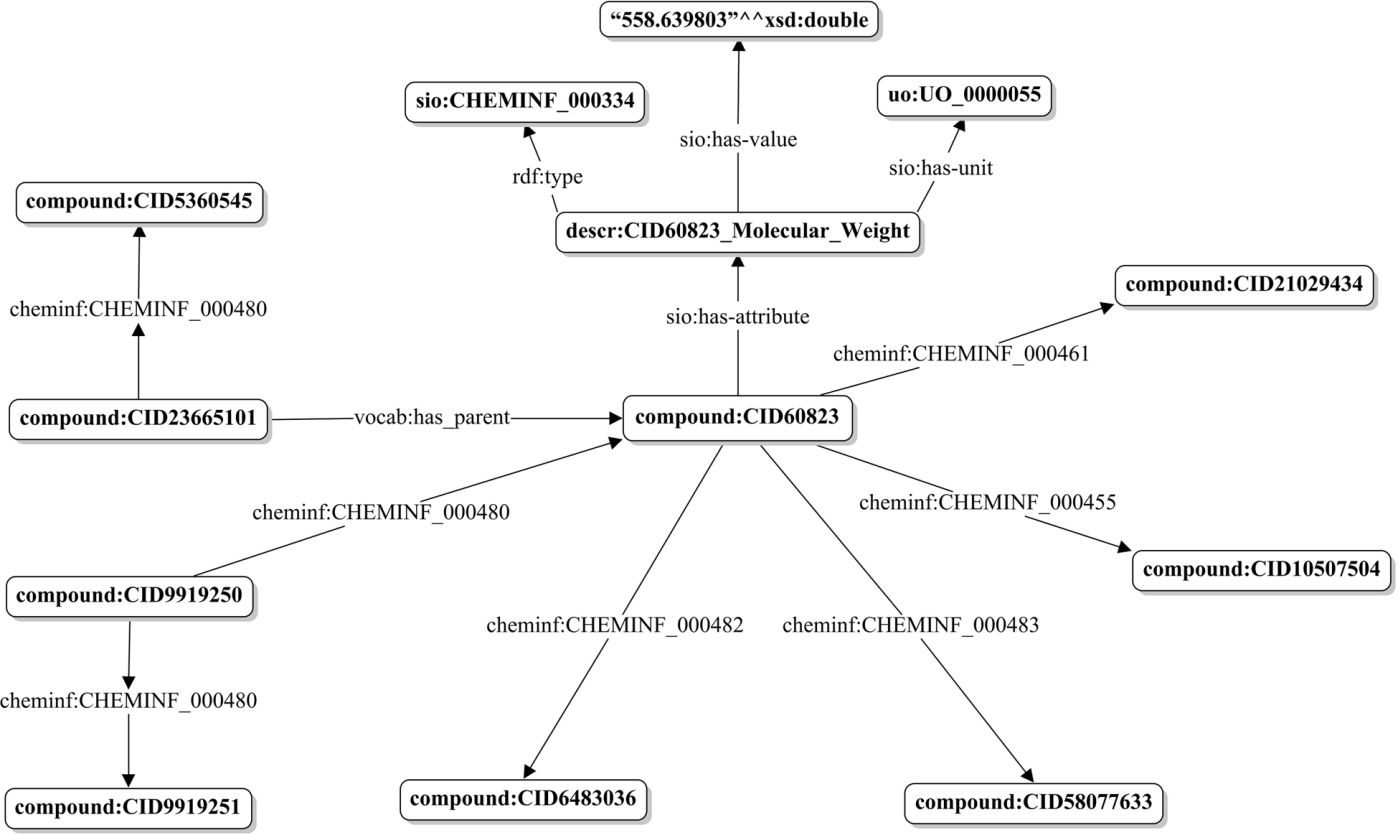
RDF diagram representing the calculated attributes of CID60823, and its interconnections with other compounds.

All PubChem compounds are associated with computed compound-centric descriptors that are calculated by PubChem. For instance, the molecular weight of compound CID60823 is an attribute instance, and the association is exposed as:

$$\begin{aligned}&\quad \tt{Compound{:}CID60823\,\, sio{:}has}{\texttt{-}}\tt{ attribute} \\ &\tt{descr{:}CID60823\_Molecular\_Weight.} \end{aligned}$$

where the calculated value, unit, and type of the given chemical descriptor are exposed in the descriptor subdomain (see Figure 3).

PubChem chemical structure processing identifies structural overlaps and correlations under different circumstances. If a compound has more than one separate covalent unit, it is considered a mixture; if one of the covalent units can be considered to be a ‘parent’ compound (see below), it is considered to be a salt mixture. One or more component compounds are associated with the corresponding mixture. Beyond the chemical composition relationship, PubChem designates an identity grouping relationship between compounds, called a Compound Identity Group (CIG). This grouping information for related compounds is utilized to help associate various isotopic and stereo isoforms provided by PubChem contributors. To implement this, several different levels of ‘sameness’ are considered: *same*-*stereochemistry* (isotope-form can vary), *same*-*isotope* (stereo-form can vary), and *same*-*connectivity* (isotope- and stereo-form can vary). In addition, the similarity neighboring between compounds are also incorporated in PubChem Compound based on different 2-D/3-D structural features [44]. The relations linking two compounds have been exposed as object properties in CHEMINF, with well-defined domain, range, and axioms. As an example, CID60823 is a component in over 400 mixture compounds. The chemical composition relations for one of these mixtures are expressed using predicate $\tt{cheminf:CHEMINF\_000480 }$ (see Table 2) as follows (see Figure 3):

$$\begin{aligned}& \tt{compound{:}CID9919250 \,\,cheminf{:}CHEMINF\_000480} \\ &\quad \tt{compound{:}CID60823, \,\,compound{:}CID9919251.} \end{aligned}$$

All components are acid/base neutralized as-is possible. If a component contains a super majority (≥70%) of all heavy (non-hydrogen) atoms across all unique components of a mixture and if that component has at least one carbon atom, it is designated as the parent component. In the above case, CID9919250 does not have a parent component. Another compound, CID23665101, has the parent component CID60823, and the other component CID5360545 is the salt counter-ion (non-parent component):

$$\begin{aligned}& \tt{compound{:}CID23665101\,\, vocab{:}has\_parent \,\,compound{:}CID60823;} \\&\quad \tt{cheminf{:}CHEMINF\_000480 \,\,compound{:}CID5360545.} \end{aligned}$$

A compound is the parent of itself, its acid/base conjugates, and its salt-form variations. As such, the parent designation is helpful to aggregate the neutralized-form of a chemical structure with their salt-form or ionized-form variations, as is custom to do in bioactivity data analysis of organic chemicals, where the salt component is often assumed to not participate in the biological activity. Furthermore, according to the ontological representation:

$$\tt{vocab{:}has\_parent\,\, rdfs{:}subPropertyOf\,\, cheminf{:}CHEMINF\_000480.}$$

the following statement (inferred) is also true:

$$\tt{compound{:}CID23665101 \,\,cheminf{:}CHEMINF\_000480 \,\,compound{:}CID60823.}$$

Although the inferred statements are not explicitly stated in the dataset, they can be queried in the same way as the asserted statements when the RDF schema is recognized as the rule set by the reasoning engine.

Moreover, CID60823 is an isotopologue of CID10507504, and it is a stereoisomer of CID21029434. The CIG relations are expressed as follows (see Table 2; Figure 3):

$$\begin{aligned}& \tt{compound{:}CID60823} \\&\quad \tt{cheminf{:}CHEMINF\_000455\,\, compound{:}CID10507504;} \\&\quad \tt{cheminf{:}CHEMINF\_000461 \,\,compound{:}CID21029434.} \end{aligned}$$

again according to the ontological definition:

$$\begin{aligned}&\tt{cheminf{:}CHEMINF\_000455} \\ &\quad \tt{rdfs{:}subPropertyOf \,\,cheminf{:}CHEMINF\_000462.} \\& \tt{cheminf{:}CHEMINF\_000461} \\ &\quad \tt{rdfs{:}subPropertyOf \,\,cheminf{:}CHEMINF\_000462.} \end{aligned}$$

the following statements (inferred) are also true:

$$\begin{aligned} &\tt{compound{:}CID60823} \\&\quad \tt{cheminf{:}CHEMINF\_000462\,\, compound{:}CID10507504;} \\ &\quad \tt{cheminf{:}CHEMINF\_000462 \,\,compound{:}CID21029434} \end{aligned}$$

Last but not least, CID60823 has over 800 structural similarity neighbors assigned by PubChem chemical structure processing. The similarity neighboring relations can be expressed using predicates $\tt{cheminf{:}CHEMINF\_000482}$ and $\tt{cheminf{:}CHEMINF\_000483}$ (see Table 2) as follows, showing a single example for each of the two similarity types for CID60823 (see Figure 3):

$$\begin{aligned} &\tt{compound{:}CID60823} \\ &\quad \tt{cheminf{:}CHEMINF\_000482\,\, compound{:}CID10030610;} \\ &\quad \tt{cheminf{:}CHEMINF\_000483 \,\,compound{:}CID11330946.} \end{aligned}$$

### Compound neighboring

**Figure 4:**
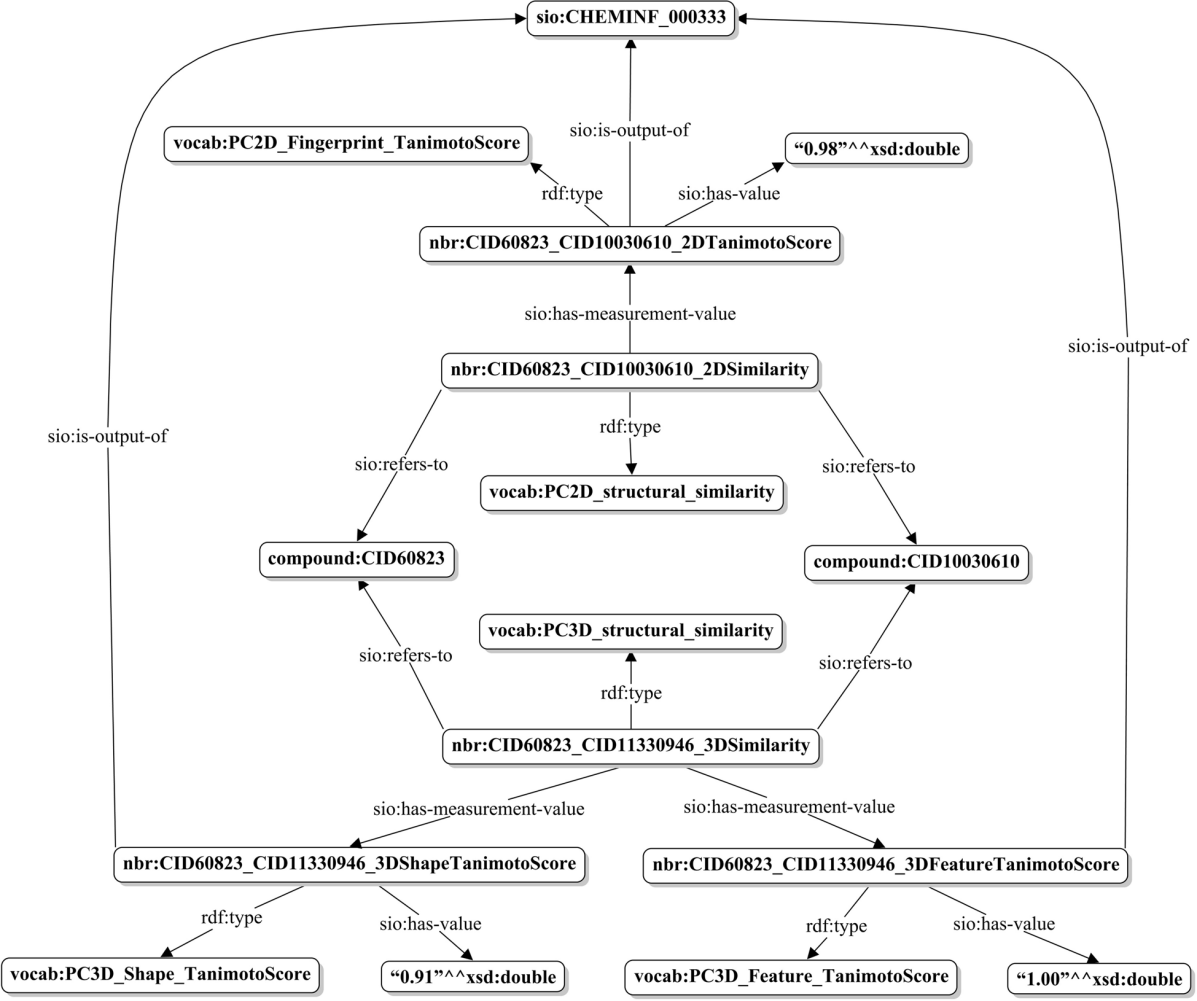
RDF diagram representing PubChem 2-/3-D similarity neighboring and score.

PubChem 2-D similarity neighbors are determined based on Tanimoto scores ≥0.9, which are calculated using binary substructure fingerprints (881 bits in length) [45]. PubChem 3-D similarity neighbors are determined based on two 3-D Tanimoto scores, calculated using 3-D conformers which are pre-computed for more than 90% of the PubChem compound records [46]. The two complementary 3-D Tanimoto scores are calculated for conformer neighbor pairs based on shape-optimized structural overlap and Gaussian-function aided volume integration: 3-D Shape Tanimoto (ST) and 3-D Color Tanimoto (CT) [44]. If two compounds have pharmacophore features (e.g., hydrogen bond acceptors), a threshold of ST ≥ 0.80 and CT ≥ 0.50 is used to determine the 3-D similarity neighboring; otherwise, a threshold of ST ≥ 0.93 is used if neither compound has pharmacophore features. Although one RDF triple can be used to link two compounds according to their 2-D or 3-D similarity neighboring, the quantitative similarity scores cannot be expressed in the same triple. Hence, a set of triples were designed by instantiating similarity neighbor associations and score entities, in order to capture this knowledge (see Figure 4):

$$\begin{aligned}& \tt{nbr{:}CID60823\_CID10030610\_2DSimilarity} \\&\quad \tt{sio{:}has}{\texttt{-}}\tt{measurement}{\texttt{-}}\tt{value} \\ &\quad \tt{nbr{:}CID60823\_CID10030610\_2DTanimotoScore;} \\ &\quad \tt{sio{:}refers}{\texttt{-}}\tt{to \,\,compound{:}CID10030610, compound{:}CID60823;} \\ &\quad \tt{rdf{:}type \,\, vocab{:}PC2D\_structural\_similarity.} \end{aligned}$$

The structural similarity is a subclass of $\tt{sio:association}$, utilized to annotate the relation between two entities. This strategy for RDF n-ary representation of relational associations between two or more entities has been widely adopted. Gene-disease associations and protein–protein interactions have been successfully annotated in a similar manner, which were deposited in Nanopub.org [47]. The quantitative score assessing the structural similarity is expressed as:

$$\begin{aligned}& \tt{nbr{:}CID60823\_CID10030610\_2DTanimotoScore} \\&\quad \tt{sio{:}has}{\texttt{-}}\tt{value \,\,"0.98"^{\wedge\wedge}}\tt{xsd{:}double;} \\ &\quad \tt{rdf{:}type\,\, vocab{:}PC2D\_Fingerprint\_TanimotoScore.}\\ \end{aligned}$$

The 3-D structural similarity is evaluated using two complementary 3-D Tanimoto scores (see Figure 4) and is expressed as such:

$$\begin{aligned}& \tt{nbr{:}CID60823\_CID11330946\_3DSimilarity} \\ &\quad \tt{sio{:}has}{\texttt{-}}\tt{measurement}{\texttt{-}}\tt{value} \\&\quad \tt{score{:}CID60823\_CID11330946\_3DFeatureTanimotoScore,} \\ &\quad \tt{score{:}CID60823\_CID11330946\_3DShapeTanimotoScore;} \\&\quad \tt{sio{:}refers}{\texttt{-}}\tt{to \, compound{:}CID11330946, \, compound{:}CID60823;} \\&\quad \tt {rdf{:}type vocab{:}PC3D\_structural\_similarity.} \\ & \tt{nbr{:}CID60823\_CID11330946\_3DFeatureTanimotoScore} \\&\quad \tt{sio{:}has}{\texttt{-}}\tt{ value "0.59"}^{\wedge\wedge}\tt{xsd{:}double;} \\&\quad \tt{rdf{:}type \, vocab{:}PC3D\_Feature\_TanimotoScore.} \\ & \tt{nbr{:}CID60823\_CID11330946\_3DShapeTanimotoScore} \\&\quad \tt{sio{:}has}{\texttt{-}}\tt{value\,\, "0.88"}^{\wedge\wedge}\tt{xsd{:}double;} \\&\quad \tt{rdf{:}type \, vocab{:}PC3D\_Shape\_TanimotoScore.}\\ \end{aligned}$$

In addition to semantic annotation of the quantitative similarity scores between compounds, the provenance metadata of PubChem 2-D/3-D Tanimoto scores can also be expressed using object property $\tt{sio{:}is}{\texttt{-}}\tt{output}{\texttt{-}}\tt{of}$ (see Figure 4).

### Descriptor, InChIKey, and synonym

The chemical descriptor representation consists of triples specifying the type, value, and unit associated with the chemical descriptor, as is appropriate. The following RDF statements in turtle syntax represent the description of molecular weight as a property for CID60823 (see Figure 3):

$$\begin{aligned} &\tt{descr{:}CID60823\_Molecular\_Weight} \\&\quad \tt{rdf{:}type\,\, sio{:}CHEMINF\_000334;} \\&\quad \tt{sio{:}has}{\texttt{-}}\tt{value \,\,"558.639803"}^{\wedge\wedge}\tt{xsd{:}double;} \\&\quad \tt{sio{:}has}{\texttt{-}}\,\,\tt{unit uo{:}UO\_0000055.} \end{aligned}$$

where the descriptor value conforms to the data types defined in XML schema [48]. A list of calculated chemical descriptors exposed in PubChemRDF statements is found in Table 3, and each of them is formally typed using the CHEMINF vocabulary. Representing properties in a central vocabulary such as CHEMINF enables comparison between chemical properties arising from different databases in a standardized fashion. The software used by PubChem for calculating descriptor values is also defined in CHEMINF, as shown in Table 4.

**Table 3:** Calculated chemical descriptor and the corresponding ontology term ID

Property name	Term ID	Software library
Molecular weight	CHEMINF_000334	PubChem
Molecular formula	CHEMINF_000335	
Total formal charge	CHEMINF_000336	
Mono isotopic weight	CHEMINF_000337	
Exact mass	CHEMINF_000338	
Compound identifier	CHEMINF_000140	
Covalent unit count	CHEMINF_000369	
Defined atom stereocenter count	CHEMINF_000370	
Defined bond stereocenter count	CHEMINF_000371	
Isotope atom count	CHEMINF_000372	
Heavy atom count	CHEMINF_000373	
Undefined atom stereocenter count	CHEMINF_000374	
Undefined bond stereocenter count	CHEMINF_000375	
Canonical SMILES	CHEMINF_000376	OEChem
Isomeric SMILES	CHEMINF_000379	
Preferred IUPAC name	CHEMINF_000382	LexiChem
Hydrogen bond donor count	CHEMINF_000387	Cactvs
Hydrogen bond acceptor count	CHEMINF_000388	
Rotatable bond count	CHEMINF_000389	
Structure complexity	CHEMINF_000390	
Tautomer count	CHEMINF_000391	
TPSA	CHEMINF_000392	
XLogP3	CHEMINF_000395	XLogP3
IUPAC InChI	CHEMINF_000396	InChI
IUPAC InChIKey	CHEMINF_000399	

The software library used by PubChem to calculate property values are associated with each chemical property.

**Table 4:** Name, version, and corresponding CHEMINF term ID for software libraries used to calculate chemical properties of PubChem compound

Name	Version^a^	CHEMINF term ID
PubChem	2.1	CHEMINF_000333
OEChem	1.9.0	CHEMINF_000429
LexiChem	2.2.0	CHEMINF_000384
Cactvs	3.408	CHEMINF_000386
XLogP3	3.0	CHEMINF_000394
InChI	1.0.4	CHEMINF_000398

^a^Please note that these versions will change as a function of software updates.

Calculated InChIKey and depositor-provided synonyms were exposed in separate subdomains. The associated CIDs of a given InChIKey and synonym are linked through predicate $\tt{sio{:}is}{\texttt{-}}\tt{attribute}{\texttt{-}}\tt{of}$ (see Figure 1) within their subdomains:

$$\begin{aligned} &\tt{inchikey{:}XUKUURHRXDUEBC}{\texttt{-}}\tt{KAYWLYCHSA}{\texttt{-}}\tt{N} \\ &\quad \tt{sio{:}is}{\texttt{-}}\tt{attribute}{\texttt{-}}\tt{of\,\, compound{:}CID60823.} \\& \tt{syno{:}MD5\_9a05646d461669f86de312d88ab5748a} \\&\quad \tt{sio{:}is}{\texttt{-}}\tt{attribute}{\texttt{-}}\tt{of\,\, compound{:}CID60823.} \end{aligned}$$

It is noteworthy that although depositor-provided synonyms are attributes of both PubChem substances and compounds, not all of the synonyms of a given substance are automatically assigned as the synonyms of the corresponding compound. A crowdsourcing-based voting mechanism is implemented to filter out anomalous name/structure associations and to resolve conflicts of name/structure associations from various data sources. So if the majority votes as per the algorithm agree on a given name/structure association, there would be two triples specifying the link between the synonym and substance (in the substance subdomain), as well as the link between the synonym and compound (in the synonym subdomain). Otherwise, only one triple would be available, linking the synonym and substance (in the substance subdomain).

The type and value of a given synonym are exposed as well (see Figure 1). In order to maximally leverage metadata for chemical name searches, different subtypes of synonyms were specified, including the chemical abstract service (CAS) registry number, unique ingredient identifiers (UNIIs), drug trade names, international nonproprietary names (INNs), and so on (see Table 5). The subtypes of the depositor-provided identifiers as a substance-centric descriptor were also specified to some extent. Since there are hundreds of types of depositor-provided identifiers and many of these are not frequently used, it would be unrealistic to annotate all of them. Therefore, only several subtypes of depositor-provided identifiers have been explicitly distinguished, and the rest of them were typed as validated chemical database identifiers (CHEMINF_000467) (see Table 5). Annotating the types of synonyms and identifiers allows data items to be grouped at a semantic level rather than only at a syntactic level.

**Table 5:** The types and corresponding CHEMINF term ID of the depositor-provided synonyms and identifiers

Database identifier	CHEMINF term ID
ChEMBL identifier	CHEMINF_000412
KEGG identifier	CHEMINF_000409
Human Metabolome Database identifier	CHEMINF_000408
ChemSpider identifier	CHEMINF_000405
ChEBI identifier	CHEMINF_000407
DrugBank identifier	CHEMINF_000406
CAS registry number	CHEMINF_000446
EC number^a^	CHEMINF_000447
RTECS number^b^	CHEMINF_000566
LipidMaps identifier	CHEMINF_000564
National service center number	CHEMINF_000565
Unique ingredient identifier	CHEMINF_000563
Validated chemical database identifier^c^	CHEMINF_000467
Drug trade name	CHEMINF_000561
International nonproprietary name	CHEMINF_000562
PubChem depositor-supplied name	CHEMINF_000339

^a^A seven-digit identifier for chemical substances for regulatory purposes within the European Union.

^b^Identifying numbers used in the Registry of Toxic Effects of Chemical Substances (RTECS) database of toxicity information.

^c^ Identifying descriptor is the superclass of other identifier types in the table.

In addition, whenever an InChIKey or a synonym represents a chemical structure that belongs to a Medical Subject Headings (MeSH) concept or an ATC concept, its major topic is annotated using predicate $\tt{dcterms:subject}$ (see Figure 1):

$$\begin{aligned} &\tt{inchikey{:}XUKUURHRXDUEBC}{\texttt{-}}\tt{KAYWLYCHSA}{\texttt{-}}\tt{N} \\&\quad \tt{dcterms{:}subject \,\,mesh{:}M0179294.} \\ &\tt{syno{:}MD5\_9a05646d461669f86de312d88ab5748a} \\ &\quad \tt{dcterms{:}subject \,\,mesh{:}M0179294.} \\& \tt{syno{:}MD5\_9a05646d461669f86de312d88ab5748a} \\ &\quad \tt{dcterms{:}subject \,\,concept{:}ATC\_C10AA05.} \end{aligned}$$

### Data sources

PubChem substance contents are provided by a variety of data sources. Exposing provenance and attribution metadata is helpful to evaluate the reliability and creditability of data sources, as well as to integrate the diverse information from them. The provenance for SID103554720 is described as follows:

$$\tt{substance{:}SID103554720 \,\,dcterms{:}source \,\,source{:}ChEMBL.}$$

where $\tt{source{:}ChEMBL}$ is represented as an instance of $\tt{dcterms{:}Dataset}$, and the title and alternative names (if possible) for the dataset was exposed through predicate $\tt{dcterms{:}title}$ and $\tt{dcterms{:}alternative}$.

In order to guide better navigation through data sources, PubChem allows depositors to categorize the type of information they provide or that their resource contains. This is exposed as the “substance categorization classification”. The categories may be either topic-related such as biological properties, chemical reactions, metabolic pathways, physical properties, protein 3D structures, theoretical properties, and toxicology; or depositor identity-related such as imaging agents, journal publishers, molecular libraries screening center network, NIH substance repository, and substance vendors [49]. A single data source may be attributed to multiple categories. The predicate $\tt{dcterms{:}subject}$ can be used to tag a data source with a specific topic, subsequently, to classify the data source into corresponding categories. The dataset topic in each category is an instance of $\tt{skos{:}concept}$, and is in a concept scheme named Substance Categorization Classification. The corresponding RDF graph describing data provenance is depicted in Figure 2.

## Utility and discussion

The semantic relations between PubChem Compound and Substance provide a way to aggregate and interlink information from different data sources based on the same canonical representation of a chemical structure. For instance, CID60823 (atorvastatin) refers to a standardized chemical structure derived from several Substance records including: SID26697365 deposited by ChEBI, which can be related to the structure-based classification according to the ChEBI ontology; SID51091801 deposited by Kyoto Encyclopedia of Genes and Genomes (KEGG), which contains information on biological pathways and biomolecular interactions; SID822166 deposited by Molecular Modeling Database (MMDB), which has protein-bound 3D structure information; SID135019185 deposited by ChemIDplus, which correlates toxicology and safety references to the given chemical structure; and SID103554720 deposited by ChEMBL, which associates bioactivity profiles to the given chemical structure. As a result, the resources across chemical, biological, and life science domain can be interlinked for CID60823. If the RDF statements were loaded into a triple store with SPARQL query interface, the following SPARQL query can be used to retrieve all of the substances and data sources associated with CID60823:

$$\begin{aligned} &\tt{SELECT\,\, DISTINCT\,\, ?substance \,\,?source} \\ &\tt{WHERE\,\, \{} \\ &\quad \tt{?substance\,\, sio{:}CHEMINF\_000477\,\, compound{:}CID60823.} \\&\quad \tt{?substance \,\,dcterms{:}source\,\, ?source.} \\& \tt{\}} \end{aligned}$$

Once integrated, the domain knowledge can be shared across data sources. For instance, the pharmacological roles defined in ChEBI ontology can be used to annotate a given chemical found in PDB crystal structure [PDB:1HWK]:

$$\begin{aligned} &\tt{PREFIX\,\, obov{:} <http{:}//purl.obolibrary.org/obo\#>} \\ &\tt{SELECT\,\, DISTINCT\,\, ?rolelabel} \\ &\tt{WHERE \,\,\{} \\&\quad \tt{?substance\,\, pdbo{:}link\_to\_pdb \,\,pdbr{:}1HWK.} \\ &\quad \tt{?substance\,\, rdf{:}type\,\, ?chebi.} \\ &\quad \tt{?chebi\,\, rdfs{:}subClassOf\, [ \,a\,\, owl{:}Restriction;} \\&\qquad \tt{owl{:}onProperty \,\,obov{:}has\_role;} \\ &\qquad \tt{owl{:}someValuesFrom\,\, ?role \,].} \\&\quad \tt{?role\,\, rdfs{:}label\,\, ?rolelabel.} \\& \tt{\}} \end{aligned}$$

The query returned two different pharmacological roles, which are “antilipemic drug”, and “hydroxymethylglutaryl-CoA reductase inhibitor”. In order to perform the query in the local computing resources, both PubChemRDF data and ChEBI ontology should be loaded into the same RDF store.

The chemical descriptors serve as quantified attributes to describe PubChem Compound and Substance records. The PubChemRDF design utilizes object properties $\tt{sio{:}has}{\texttt{-}}\tt{attribute}$ and $\tt{sio{:}has}{\texttt{-}}\tt{value}$ to specify the relations between the chemical entities and the associated descriptors. SIO is developed to support knowledge representation and reasoning in the scientific research, and the same design pattern has been implemented in the Bio2RDF mash-up system [22, 23] and the Semantic Automated Discovery and Integration (SADI) [50, 51] web service. Re-use of such design patterns across multiple Semantic Web offerings reduces the effort it takes to construct federated queries. The data consumers can refine a collection of PubChem Compound or Substance records according to the values of a given chemical descriptor. For instance, a PubChemRDF user can search for the PubChem compounds that belong to non-steroidal anti-inflammatory drugs (NSAIDs) defined in ChEBI, and have molecular weight less than 200:

$$\begin{aligned} & \tt{PREFIX \,\,obov{:}\,\, <http{:}//purl.obolibrary.org/obo\#>} \\ & \tt{SELECT\,\, distinct\,\, ?compound} \\ &\tt{WHERE \ \{} \\ &\tt{?compound \,\,rdf{:}type\,\, ?chebi.} \\ &\tt{?chebi \,\,rdfs{:}subClassOf \,[\, a\,\, owl{:}Restriction;} \\ &\tt{owl{:}onProperty \,\,obov{:}has\_role;} \\ &\tt{owl{:}someValuesFrom \,\,obo{:}CHEBI\_35475 ].} \\ &\tt{?comp \,\,sio{:}has}{\texttt{-}}\tt{attribute\,\, ?MW.} \\ &\tt{?MW\,\, rdf{:}type\,\, sio{:}CHEMINF\_000334.} \\& \tt{?MW \,\,sio{:}has}{\texttt{-}}\tt{value\,\, ?MWValue.} \\ &\tt{FILTER (\, ?MWValue < 200\,)}\\ &\tt{\}} \end{aligned}$$

The SPARQL query returned 72 different compounds listed in Additional file 1: Table S1.

In order to bridge RDF data publishing and RDF data consumption, a variety of semantic data models have been proposed for the provenance and attribution metadata. These include Nanopublication [52], Bio2RDF [53], and Open PHACTS [54] dataset provenance models. The PubChemRDF project also provides provenance and attribution metadata for various data sources, and the provenance descriptions originate and augment the Open PHACTS dataset descriptions. Since the topics used to categorize data sources in PubChem are highly domain-specific, we assigned $\tt{skos{:}Concept}$ URIs to attempt to precisely capture the categorization of PubChem data sources. These metadata can be very helpful for information retrieval and refinement. For instance, a PubChemRDF user can collect a set of PubChem substances that belong to NSAIDs defined in ChEBI and come from data sources providing protein 3-D structures:

$$\begin{aligned} &\tt{PREFIX obov{:} \,\,<http{:}//purl.obolibrary.org/obo\#>} \\ &\tt{SELECT \,\,DISTINCT \,\,?substance \,\,?source} \\ &\tt{WHERE \ \{} \\ &\tt{?substance\,\, dcterms{:}source\,\, ?source.} \\ &\tt{?source \,\,dcterms{:}subject\,\, concept{:}Protein\_3D\_Structures.} \\ &\tt{?substance\,\,rdf{:}type\,\,?chebi.} \\ &\tt{?chebi\,\,rdfs{:}subClassOf\,[\, a\,owl{:}Restriction;} \\ &\tt{owl{:}onProperty \,\,obov{:}has\_role;} \\ &\tt{owl{:}someValuesFrom\,\,obo{:}CHEBI\_35475 \,].} \\ &\tt{\}} \end{aligned}$$

The query return 115 different substances associated with their data sources, most of which were deposited by the Molecular Modeling Database (MMDB). The complete list is available in Additional file 1: Table S2.

The PubChemRDF project allows maximal flexibility to cross-reference a PubChem Substance record with other data sources. For instance, SID103554720 is interchangeable to an external RDF-based resource, and the fact is declared as a RDF triple:

$$\tt{substance{:}SID103554720\,\,skos{:}exactMatchlinkedchem{:}CHEMBL1487.}$$

where the predicate $\tt{skos{:}exactMatch}$ was also employed by the Open PHACTS project for cross-reference. Cross-linking to other RDF-based resources entails federated queries over other remote SPARQL endpoints. For instance, the following federated query can be used to search the Uppsala SPARQL endpoint for ChEMBL RDF triples [27] related to SID103554720:

$$\begin{aligned} &\tt{PREFIX\,\,onto{:}\,\,<http{:}//rdf.farmbio.uu.se/chembl/onto/\#>} \\ &\tt{SELECT\,\,DISTINCT\,\,?rel\,\,?value\,\,?unit\,\,?label} \\ &\tt{WHERE \ \{} \\ &\tt{substance{:}SID103554720\,\,skos{:}exactMatch\,\,?chembl.} \\ &\tt{SERVICE\,\,<http{:}//rdf.farmbio.uu.se/chembl/sparql>} \\ &\tt{WHERE\ \{} \\ &\tt{?chembl\,\,owl{:}equivalentClass\,\,?mol.} \\ &\tt{?act\,\,onto{:}forMolecule\,\,?mol.} \\ &\tt{?act\,\,onto{:}relation\,\,?rel.} \\ &\tt{?act\,\,onto{:}standardValue\,\,?value.} \\ &\tt{?act\,\,onto{:}standardUnits\,\,?unit.} \\ &\tt{?act\,\,onto{:}type\,\,?type.} \\ &\tt{?act\,\,onto{:}onAssay\,\,?assay.} \\ &\tt{?assay\,\,rdfs{:}label\,\,?label.} \\ &\tt{\}} \\ &\tt{\}} \end{aligned}$$

The query returned 97 different bioactivities associated with corresponding ChEMBL assays. The complete list of query results is available in Additional file 1: Table S3.

## Conclusion

As described above, with the goal of semantically describing the information available in the PubChem archive, pre-existing ontological frameworks were used, rather than creating new ones. Semantic relationships between compounds and substances, chemical descriptors associated with compounds and substances, interrelationships between chemicals, as well as provenance and attribute metadata of substances were described. Future PubChemRDF papers will cover the semantic description of additional PubChem information such as bioactivity data and cross-references to proteins, genes, patents, or biomedical literature, among others.

PubChemRDF exposes data content that may not be available in any of currently existing RDF-based cheminformatics and bioinformatics resources, and it is designed to be highly compatible and consistent with them by incorporating the commonly used ontologies and vocabularies. All of the PubChemRDF URIs are dereferencable, once the exposed URIs are cross-linked by other RDF-based resources, the semantic integration should be fairly easy for end users. When considered in a wider context, there may be many promising benefits to integrating a semantic description of the PubChem chemical knowledgebase with other semantically described biological and life science domain knowledge bases. Semantic annotation of the PubChem Compound and Substance data systems works towards a machine-understandable knowledge representation, and helps pave the way to more automated and holistic data integration of scientific information. Given a collection of RDF statements describing the types and relations based on a set of formal ontologies, it is feasible to expose PubChem chemical resources to cross-domain queries, and more cross-site interoperable web applications. In addition, PubChemRDF provides a new ability for researchers to utilize schema-less data systems and so-called RDF-triple stores with SPARQL query engines to analyze data available within PubChem using local computing resources.

## Availability

The dataset is publically available without license restrictions, and it can be either accessed through REST interface (documented at: https://pubchem.ncbi.nlm.nih.gov/rdf/) or downloaded at: ftp://ftp.ncbi.nlm.nih.gov/pubchem/RDF.

## Additional file

**Additional file 1.** The supporting information for the paper entitled: PubChemRDF: towards the semantic annotation of PubChem compound and substance databases.

## Abbreviations

ChEBI: Chemical Entities of Biological Interest
CHEMINF: Chemical Information Ontology
CID: PubChem compound identifier
EBI: European Bioinformatics Institute
INN: international nonproprietary names
NCBI: National Center for Biotechnology Information
OWL: Web Ontology Language
RDF: Resource Description Framework
SID: PubChem substance identifier
SIO: semanticscience integrated ontology
URI: uniform resource identifier
UNII: unique ingredient identifier
XML: eXtenstible markup language
