# Supplementary material for: PubChemRDF: towards the semantic annotation of PubChem compound and substance databases
Source: J Cheminform. 2015 Jul 14;7:34. doi: 10.1186/s13321-015-0084-4 (PMC4500850; doi:10.1186/s13321-015-0084-4)
Supplement: Supplementary file 1 — Additional file 1. The supporting information for the paper entitled: PubChemRDF: towards the semantic annotation of PubChem compound and substance databases. [file 13321_2015_84_MOESM1_ESM.docx]

**Supporting Information for**

**PubChemRDF: Towards the Semantic Annotation of PubChem Compound and Substance Databases**

Authors:

Gang Fu,^1^ Colin Batchelor,^2^ Michel Dumontier,^3^ Janna Hastings,^4^ Egon Willighagen,^5^ Evan Bolton^1*^

Affiliations:

^1^ National Center for Biotechnology Information, National Library of Medicine, National Institute of Health, Bethesda, MD, USA

^2^ Royal Society of Chemistry, Thomas Graham House, Cambridge, UK CB4 0WF

^3^ Stanford Center for Biomedical Informatics Research, Stanford University, Stanford, USA

^4^ European Molecular Biology Laboratory – European Bioinformatics Institute (EMBL-EBI), UK

^5^ Department of Bioinformatics - BiGCaT, NUTRIM, Maastricht University

**Table S1.** The compounds that belong to non-steroidal anti-inflammatory drugs (NSAIDs) defined in ChEBI, and have molecular weight less than 200.

| Compound |
| --- |
| http://rdf.ncbi.nlm.nih.gov/pubchem/compound/CID5281515 |
| http://rdf.ncbi.nlm.nih.gov/pubchem/compound/CID3778 |
| http://rdf.ncbi.nlm.nih.gov/pubchem/compound/CID60490 |
| http://rdf.ncbi.nlm.nih.gov/pubchem/compound/CID23693301 |
| http://rdf.ncbi.nlm.nih.gov/pubchem/compound/CID1676 |
| http://rdf.ncbi.nlm.nih.gov/pubchem/compound/CID2244 |
| http://rdf.ncbi.nlm.nih.gov/pubchem/compound/CID6009 |
| http://rdf.ncbi.nlm.nih.gov/pubchem/compound/CID5280704 |
| http://rdf.ncbi.nlm.nih.gov/pubchem/compound/CID5315615 |
| http://rdf.ncbi.nlm.nih.gov/pubchem/compound/CID439503 |
| http://rdf.ncbi.nlm.nih.gov/pubchem/compound/CID60726 |
| http://rdf.ncbi.nlm.nih.gov/pubchem/compound/CID17198 |
| http://rdf.ncbi.nlm.nih.gov/pubchem/compound/CID6335412 |
| http://rdf.ncbi.nlm.nih.gov/pubchem/compound/CID2165 |
| http://rdf.ncbi.nlm.nih.gov/pubchem/compound/CID2214 |
| http://rdf.ncbi.nlm.nih.gov/pubchem/compound/CID30951 |
| http://rdf.ncbi.nlm.nih.gov/pubchem/compound/CID2206 |
| http://rdf.ncbi.nlm.nih.gov/pubchem/compound/CID60164 |
| http://rdf.ncbi.nlm.nih.gov/pubchem/compound/CID2161 |
| http://rdf.ncbi.nlm.nih.gov/pubchem/compound/CID3335 |
| http://rdf.ncbi.nlm.nih.gov/pubchem/compound/CID4164 |
| http://rdf.ncbi.nlm.nih.gov/pubchem/compound/CID2466 |
| http://rdf.ncbi.nlm.nih.gov/pubchem/compound/CID5468 |
| http://rdf.ncbi.nlm.nih.gov/pubchem/compound/CID3474 |
| http://rdf.ncbi.nlm.nih.gov/pubchem/compound/CID5733 |
| http://rdf.ncbi.nlm.nih.gov/pubchem/compound/CID54683953 |
| http://rdf.ncbi.nlm.nih.gov/pubchem/compound/CID2581 |
| http://rdf.ncbi.nlm.nih.gov/pubchem/compound/CID5357077 |
| http://rdf.ncbi.nlm.nih.gov/pubchem/compound/CID2794 |
| http://rdf.ncbi.nlm.nih.gov/pubchem/compound/CID26098 |
| http://rdf.ncbi.nlm.nih.gov/pubchem/compound/CID3059 |
| http://rdf.ncbi.nlm.nih.gov/pubchem/compound/CID73568 |
| http://rdf.ncbi.nlm.nih.gov/pubchem/compound/CID11370 |
| http://rdf.ncbi.nlm.nih.gov/pubchem/compound/CID39912 |
| http://rdf.ncbi.nlm.nih.gov/pubchem/compound/CID1983 |
| http://rdf.ncbi.nlm.nih.gov/pubchem/compound/CID3371 |
| http://rdf.ncbi.nlm.nih.gov/pubchem/compound/CID4495 |
| http://rdf.ncbi.nlm.nih.gov/pubchem/compound/CID68756 |
| http://rdf.ncbi.nlm.nih.gov/pubchem/compound/CID3033 |
| http://rdf.ncbi.nlm.nih.gov/pubchem/compound/CID4781 |
| http://rdf.ncbi.nlm.nih.gov/pubchem/compound/CID3715 |
| http://rdf.ncbi.nlm.nih.gov/pubchem/compound/CID64747 |
| http://rdf.ncbi.nlm.nih.gov/pubchem/compound/CID3308 |
| http://rdf.ncbi.nlm.nih.gov/pubchem/compound/CID77993 |
| http://rdf.ncbi.nlm.nih.gov/pubchem/compound/CID3342 |
| http://rdf.ncbi.nlm.nih.gov/pubchem/compound/CID3637398 |
| http://rdf.ncbi.nlm.nih.gov/pubchem/compound/CID3394 |
| http://rdf.ncbi.nlm.nih.gov/pubchem/compound/CID123606 |
| http://rdf.ncbi.nlm.nih.gov/pubchem/compound/CID123607 |
| http://rdf.ncbi.nlm.nih.gov/pubchem/compound/CID2151 |
| http://rdf.ncbi.nlm.nih.gov/pubchem/compound/CID6538321 |
| http://rdf.ncbi.nlm.nih.gov/pubchem/compound/CID3672 |
| http://rdf.ncbi.nlm.nih.gov/pubchem/compound/CID11954316 |
| http://rdf.ncbi.nlm.nih.gov/pubchem/compound/CID522325 |
| http://rdf.ncbi.nlm.nih.gov/pubchem/compound/CID46224594 |
| http://rdf.ncbi.nlm.nih.gov/pubchem/compound/CID656631 |
| http://rdf.ncbi.nlm.nih.gov/pubchem/compound/CID3032818 |
| http://rdf.ncbi.nlm.nih.gov/pubchem/compound/CID3825 |
| http://rdf.ncbi.nlm.nih.gov/pubchem/compound/CID54678919 |
| http://rdf.ncbi.nlm.nih.gov/pubchem/compound/CID3899 |
| http://rdf.ncbi.nlm.nih.gov/pubchem/compound/CID123619 |
| http://rdf.ncbi.nlm.nih.gov/pubchem/compound/CID119607 |
| http://rdf.ncbi.nlm.nih.gov/pubchem/compound/CID4075 |
| http://rdf.ncbi.nlm.nih.gov/pubchem/compound/CID54684141 |
| http://rdf.ncbi.nlm.nih.gov/pubchem/compound/CID23681059 |
| http://rdf.ncbi.nlm.nih.gov/pubchem/compound/CID156391 |
| http://rdf.ncbi.nlm.nih.gov/pubchem/compound/CID50294 |
| http://rdf.ncbi.nlm.nih.gov/pubchem/compound/CID6003770 |
| http://rdf.ncbi.nlm.nih.gov/pubchem/compound/CID7251185 |
| http://rdf.ncbi.nlm.nih.gov/pubchem/compound/CID1548887 |
| http://rdf.ncbi.nlm.nih.gov/pubchem/compound/CID5359476 |
| http://rdf.ncbi.nlm.nih.gov/pubchem/compound/CID5359 |

**Table S2.** The substances and their sources that providing 3-D protein structure information.

| substance | source |
| --- | --- |
| <http://rdf.ncbi.nlm.nih.gov/pubchem/substance/SID85098767> | <http://rdf.ncbi.nlm.nih.gov/pubchem/source/MMDB> |
| <http://rdf.ncbi.nlm.nih.gov/pubchem/substance/SID829042> | <http://rdf.ncbi.nlm.nih.gov/pubchem/source/MMDB> |
| <http://rdf.ncbi.nlm.nih.gov/pubchem/substance/SID832958> | <http://rdf.ncbi.nlm.nih.gov/pubchem/source/MMDB> |
| <http://rdf.ncbi.nlm.nih.gov/pubchem/substance/SID26717767> | <http://rdf.ncbi.nlm.nih.gov/pubchem/source/MMDB> |
| <http://rdf.ncbi.nlm.nih.gov/pubchem/substance/SID57410906> | <http://rdf.ncbi.nlm.nih.gov/pubchem/source/MMDB> |
| <http://rdf.ncbi.nlm.nih.gov/pubchem/substance/SID170475358> | <http://rdf.ncbi.nlm.nih.gov/pubchem/source/MMDB> |
| <http://rdf.ncbi.nlm.nih.gov/pubchem/substance/SID7885805> | <http://rdf.ncbi.nlm.nih.gov/pubchem/source/SMID> |
| <http://rdf.ncbi.nlm.nih.gov/pubchem/substance/SID136349740> | <http://rdf.ncbi.nlm.nih.gov/pubchem/source/MMDB> |
| <http://rdf.ncbi.nlm.nih.gov/pubchem/substance/SID164117525> | <http://rdf.ncbi.nlm.nih.gov/pubchem/source/MMDB> |
| <http://rdf.ncbi.nlm.nih.gov/pubchem/substance/SID81062056> | <http://rdf.ncbi.nlm.nih.gov/pubchem/source/MMDB> |
| <http://rdf.ncbi.nlm.nih.gov/pubchem/substance/SID136959398> | <http://rdf.ncbi.nlm.nih.gov/pubchem/source/MMDB> |
| <http://rdf.ncbi.nlm.nih.gov/pubchem/substance/SID223366354> | <http://rdf.ncbi.nlm.nih.gov/pubchem/source/MMDB> |
| <http://rdf.ncbi.nlm.nih.gov/pubchem/substance/SID144072068> | <http://rdf.ncbi.nlm.nih.gov/pubchem/source/MMDB> |
| <http://rdf.ncbi.nlm.nih.gov/pubchem/substance/SID7886759> | <http://rdf.ncbi.nlm.nih.gov/pubchem/source/SMID> |
| <http://rdf.ncbi.nlm.nih.gov/pubchem/substance/SID89449260> | <http://rdf.ncbi.nlm.nih.gov/pubchem/source/MMDB> |
| <http://rdf.ncbi.nlm.nih.gov/pubchem/substance/SID223259225> | <http://rdf.ncbi.nlm.nih.gov/pubchem/source/MMDB> |
| <http://rdf.ncbi.nlm.nih.gov/pubchem/substance/SID160649693> | <http://rdf.ncbi.nlm.nih.gov/pubchem/source/MMDB> |
| <http://rdf.ncbi.nlm.nih.gov/pubchem/substance/SID135659609> | <http://rdf.ncbi.nlm.nih.gov/pubchem/source/MMDB> |
| <http://rdf.ncbi.nlm.nih.gov/pubchem/substance/SID174861545> | <http://rdf.ncbi.nlm.nih.gov/pubchem/source/MMDB> |
| <http://rdf.ncbi.nlm.nih.gov/pubchem/substance/SID8027932> | <http://rdf.ncbi.nlm.nih.gov/pubchem/source/MMDB> |
| <http://rdf.ncbi.nlm.nih.gov/pubchem/substance/SID8027943> | <http://rdf.ncbi.nlm.nih.gov/pubchem/source/MMDB> |
| <http://rdf.ncbi.nlm.nih.gov/pubchem/substance/SID8027946> | <http://rdf.ncbi.nlm.nih.gov/pubchem/source/MMDB> |
| <http://rdf.ncbi.nlm.nih.gov/pubchem/substance/SID8027938> | <http://rdf.ncbi.nlm.nih.gov/pubchem/source/MMDB> |
| <http://rdf.ncbi.nlm.nih.gov/pubchem/substance/SID49738138> | <http://rdf.ncbi.nlm.nih.gov/pubchem/source/MMDB> |
| <http://rdf.ncbi.nlm.nih.gov/pubchem/substance/SID163614927> | <http://rdf.ncbi.nlm.nih.gov/pubchem/source/MMDB> |
| <http://rdf.ncbi.nlm.nih.gov/pubchem/substance/SID7885083> | <http://rdf.ncbi.nlm.nih.gov/pubchem/source/SMID> |
| <http://rdf.ncbi.nlm.nih.gov/pubchem/substance/SID24715004> | <http://rdf.ncbi.nlm.nih.gov/pubchem/source/Structural_Genomics_Consortium> |
| <http://rdf.ncbi.nlm.nih.gov/pubchem/substance/SID26512241> | <http://rdf.ncbi.nlm.nih.gov/pubchem/source/Structural_Genomics_Consortium> |
| <http://rdf.ncbi.nlm.nih.gov/pubchem/substance/SID825185> | <http://rdf.ncbi.nlm.nih.gov/pubchem/source/MMDB> |
| <http://rdf.ncbi.nlm.nih.gov/pubchem/substance/SID832225> | <http://rdf.ncbi.nlm.nih.gov/pubchem/source/MMDB> |
| <http://rdf.ncbi.nlm.nih.gov/pubchem/substance/SID26737099> | <http://rdf.ncbi.nlm.nih.gov/pubchem/source/MMDB> |
| <http://rdf.ncbi.nlm.nih.gov/pubchem/substance/SID125267498> | <http://rdf.ncbi.nlm.nih.gov/pubchem/source/MMDB> |
| <http://rdf.ncbi.nlm.nih.gov/pubchem/substance/SID164194289> | <http://rdf.ncbi.nlm.nih.gov/pubchem/source/MMDB> |
| <http://rdf.ncbi.nlm.nih.gov/pubchem/substance/SID6436673> | <http://rdf.ncbi.nlm.nih.gov/pubchem/source/MMDB> |
| <http://rdf.ncbi.nlm.nih.gov/pubchem/substance/SID24277576> | <http://rdf.ncbi.nlm.nih.gov/pubchem/source/MMDB> |
| <http://rdf.ncbi.nlm.nih.gov/pubchem/substance/SID53786691> | <http://rdf.ncbi.nlm.nih.gov/pubchem/source/MMDB> |
| <http://rdf.ncbi.nlm.nih.gov/pubchem/substance/SID99206237> | <http://rdf.ncbi.nlm.nih.gov/pubchem/source/MMDB> |
| <http://rdf.ncbi.nlm.nih.gov/pubchem/substance/SID111123595> | <http://rdf.ncbi.nlm.nih.gov/pubchem/source/MMDB> |
| <http://rdf.ncbi.nlm.nih.gov/pubchem/substance/SID135653203> | <http://rdf.ncbi.nlm.nih.gov/pubchem/source/MMDB> |
| <http://rdf.ncbi.nlm.nih.gov/pubchem/substance/SID8027940> | <http://rdf.ncbi.nlm.nih.gov/pubchem/source/MMDB> |
| <http://rdf.ncbi.nlm.nih.gov/pubchem/substance/SID24771121> | <http://rdf.ncbi.nlm.nih.gov/pubchem/source/MMDB> |
| <http://rdf.ncbi.nlm.nih.gov/pubchem/substance/SID57578122> | <http://rdf.ncbi.nlm.nih.gov/pubchem/source/MMDB> |
| <http://rdf.ncbi.nlm.nih.gov/pubchem/substance/SID84982119> | <http://rdf.ncbi.nlm.nih.gov/pubchem/source/MMDB> |
| <http://rdf.ncbi.nlm.nih.gov/pubchem/substance/SID117688215> | <http://rdf.ncbi.nlm.nih.gov/pubchem/source/MMDB> |
| <http://rdf.ncbi.nlm.nih.gov/pubchem/substance/SID144186704> | <http://rdf.ncbi.nlm.nih.gov/pubchem/source/MMDB> |
| <http://rdf.ncbi.nlm.nih.gov/pubchem/substance/SID174861573> | <http://rdf.ncbi.nlm.nih.gov/pubchem/source/MMDB> |
| <http://rdf.ncbi.nlm.nih.gov/pubchem/substance/SID223404048> | <http://rdf.ncbi.nlm.nih.gov/pubchem/source/MMDB> |
| <http://rdf.ncbi.nlm.nih.gov/pubchem/substance/SID249814731> | <http://rdf.ncbi.nlm.nih.gov/pubchem/source/MMDB> |
| <http://rdf.ncbi.nlm.nih.gov/pubchem/substance/SID11532417> | <http://rdf.ncbi.nlm.nih.gov/pubchem/source/MMDB> |
| <http://rdf.ncbi.nlm.nih.gov/pubchem/substance/SID17436465> | <http://rdf.ncbi.nlm.nih.gov/pubchem/source/MMDB> |
| <http://rdf.ncbi.nlm.nih.gov/pubchem/substance/SID103905803> | <http://rdf.ncbi.nlm.nih.gov/pubchem/source/MMDB> |
| <http://rdf.ncbi.nlm.nih.gov/pubchem/substance/SID131500217> | <http://rdf.ncbi.nlm.nih.gov/pubchem/source/MMDB> |
| <http://rdf.ncbi.nlm.nih.gov/pubchem/substance/SID131500220> | <http://rdf.ncbi.nlm.nih.gov/pubchem/source/MMDB> |
| <http://rdf.ncbi.nlm.nih.gov/pubchem/substance/SID144116628> | <http://rdf.ncbi.nlm.nih.gov/pubchem/source/MMDB> |
| <http://rdf.ncbi.nlm.nih.gov/pubchem/substance/SID144186966> | <http://rdf.ncbi.nlm.nih.gov/pubchem/source/MMDB> |
| <http://rdf.ncbi.nlm.nih.gov/pubchem/substance/SID144220459> | <http://rdf.ncbi.nlm.nih.gov/pubchem/source/MMDB> |
| <http://rdf.ncbi.nlm.nih.gov/pubchem/substance/SID174861501> | <http://rdf.ncbi.nlm.nih.gov/pubchem/source/MMDB> |
| <http://rdf.ncbi.nlm.nih.gov/pubchem/substance/SID7888299> | <http://rdf.ncbi.nlm.nih.gov/pubchem/source/SMID> |
| <http://rdf.ncbi.nlm.nih.gov/pubchem/substance/SID7890920> | <http://rdf.ncbi.nlm.nih.gov/pubchem/source/SMID> |
| <http://rdf.ncbi.nlm.nih.gov/pubchem/substance/SID7887502> | <http://rdf.ncbi.nlm.nih.gov/pubchem/source/SMID> |
| <http://rdf.ncbi.nlm.nih.gov/pubchem/substance/SID7889413> | <http://rdf.ncbi.nlm.nih.gov/pubchem/source/SMID> |
| <http://rdf.ncbi.nlm.nih.gov/pubchem/substance/SID85098774> | <http://rdf.ncbi.nlm.nih.gov/pubchem/source/MMDB> |
| <http://rdf.ncbi.nlm.nih.gov/pubchem/substance/SID827876> | <http://rdf.ncbi.nlm.nih.gov/pubchem/source/MMDB> |
| <http://rdf.ncbi.nlm.nih.gov/pubchem/substance/SID832709> | <http://rdf.ncbi.nlm.nih.gov/pubchem/source/MMDB> |
| <http://rdf.ncbi.nlm.nih.gov/pubchem/substance/SID837582> | <http://rdf.ncbi.nlm.nih.gov/pubchem/source/MMDB> |
| <http://rdf.ncbi.nlm.nih.gov/pubchem/substance/SID8027112> | <http://rdf.ncbi.nlm.nih.gov/pubchem/source/MMDB> |
| <http://rdf.ncbi.nlm.nih.gov/pubchem/substance/SID57280050> | <http://rdf.ncbi.nlm.nih.gov/pubchem/source/MMDB> |
| <http://rdf.ncbi.nlm.nih.gov/pubchem/substance/SID85786798> | <http://rdf.ncbi.nlm.nih.gov/pubchem/source/MMDB> |
| <http://rdf.ncbi.nlm.nih.gov/pubchem/substance/SID99206240> | <http://rdf.ncbi.nlm.nih.gov/pubchem/source/MMDB> |
| <http://rdf.ncbi.nlm.nih.gov/pubchem/substance/SID152137967> | <http://rdf.ncbi.nlm.nih.gov/pubchem/source/MMDB> |
| <http://rdf.ncbi.nlm.nih.gov/pubchem/substance/SID223259191> | <http://rdf.ncbi.nlm.nih.gov/pubchem/source/MMDB> |
| <http://rdf.ncbi.nlm.nih.gov/pubchem/substance/SID225144516> | <http://rdf.ncbi.nlm.nih.gov/pubchem/source/MMDB> |
| <http://rdf.ncbi.nlm.nih.gov/pubchem/substance/SID8027936> | <http://rdf.ncbi.nlm.nih.gov/pubchem/source/MMDB> |
| <http://rdf.ncbi.nlm.nih.gov/pubchem/substance/SID8027956> | <http://rdf.ncbi.nlm.nih.gov/pubchem/source/MMDB> |
| <http://rdf.ncbi.nlm.nih.gov/pubchem/substance/SID8027959> | <http://rdf.ncbi.nlm.nih.gov/pubchem/source/MMDB> |
| <http://rdf.ncbi.nlm.nih.gov/pubchem/substance/SID7887044> | <http://rdf.ncbi.nlm.nih.gov/pubchem/source/SMID> |
| <http://rdf.ncbi.nlm.nih.gov/pubchem/substance/SID7889660> | <http://rdf.ncbi.nlm.nih.gov/pubchem/source/SMID> |
| <http://rdf.ncbi.nlm.nih.gov/pubchem/substance/SID14720379> | <http://rdf.ncbi.nlm.nih.gov/pubchem/source/SGCOxCompounds> |
| <http://rdf.ncbi.nlm.nih.gov/pubchem/substance/SID85098788> | <http://rdf.ncbi.nlm.nih.gov/pubchem/source/MMDB> |
| <http://rdf.ncbi.nlm.nih.gov/pubchem/substance/SID6435989> | <http://rdf.ncbi.nlm.nih.gov/pubchem/source/MMDB> |
| <http://rdf.ncbi.nlm.nih.gov/pubchem/substance/SID832223> | <http://rdf.ncbi.nlm.nih.gov/pubchem/source/MMDB> |
| <http://rdf.ncbi.nlm.nih.gov/pubchem/substance/SID8027945> | <http://rdf.ncbi.nlm.nih.gov/pubchem/source/MMDB> |
| <http://rdf.ncbi.nlm.nih.gov/pubchem/substance/SID8027950> | <http://rdf.ncbi.nlm.nih.gov/pubchem/source/MMDB> |
| <http://rdf.ncbi.nlm.nih.gov/pubchem/substance/SID8027958> | <http://rdf.ncbi.nlm.nih.gov/pubchem/source/MMDB> |
| <http://rdf.ncbi.nlm.nih.gov/pubchem/substance/SID17422208> | <http://rdf.ncbi.nlm.nih.gov/pubchem/source/MMDB> |
| <http://rdf.ncbi.nlm.nih.gov/pubchem/substance/SID24277577> | <http://rdf.ncbi.nlm.nih.gov/pubchem/source/MMDB> |
| <http://rdf.ncbi.nlm.nih.gov/pubchem/substance/SID26718878> | <http://rdf.ncbi.nlm.nih.gov/pubchem/source/MMDB> |
| <http://rdf.ncbi.nlm.nih.gov/pubchem/substance/SID53812364> | <http://rdf.ncbi.nlm.nih.gov/pubchem/source/MMDB> |
| <http://rdf.ncbi.nlm.nih.gov/pubchem/substance/SID56459035> | <http://rdf.ncbi.nlm.nih.gov/pubchem/source/MMDB> |
| <http://rdf.ncbi.nlm.nih.gov/pubchem/substance/SID74382991> | <http://rdf.ncbi.nlm.nih.gov/pubchem/source/MMDB> |
| <http://rdf.ncbi.nlm.nih.gov/pubchem/substance/SID103771594> | <http://rdf.ncbi.nlm.nih.gov/pubchem/source/MMDB> |
| <http://rdf.ncbi.nlm.nih.gov/pubchem/substance/SID103771601> | <http://rdf.ncbi.nlm.nih.gov/pubchem/source/MMDB> |
| <http://rdf.ncbi.nlm.nih.gov/pubchem/substance/SID137292101> | <http://rdf.ncbi.nlm.nih.gov/pubchem/source/MMDB> |
| <http://rdf.ncbi.nlm.nih.gov/pubchem/substance/SID137292104> | <http://rdf.ncbi.nlm.nih.gov/pubchem/source/MMDB> |
| <http://rdf.ncbi.nlm.nih.gov/pubchem/substance/SID135653204> | <http://rdf.ncbi.nlm.nih.gov/pubchem/source/MMDB> |
| <http://rdf.ncbi.nlm.nih.gov/pubchem/substance/SID160661709> | <http://rdf.ncbi.nlm.nih.gov/pubchem/source/MMDB> |
| <http://rdf.ncbi.nlm.nih.gov/pubchem/substance/SID160661710> | <http://rdf.ncbi.nlm.nih.gov/pubchem/source/MMDB> |
| <http://rdf.ncbi.nlm.nih.gov/pubchem/substance/SID163726886> | <http://rdf.ncbi.nlm.nih.gov/pubchem/source/MMDB> |
| <http://rdf.ncbi.nlm.nih.gov/pubchem/substance/SID174861550> | <http://rdf.ncbi.nlm.nih.gov/pubchem/source/MMDB> |
| <http://rdf.ncbi.nlm.nih.gov/pubchem/substance/SID7888379> | <http://rdf.ncbi.nlm.nih.gov/pubchem/source/SMID> |
| <http://rdf.ncbi.nlm.nih.gov/pubchem/substance/SID49655565> | <http://rdf.ncbi.nlm.nih.gov/pubchem/source/MMDB> |
| <http://rdf.ncbi.nlm.nih.gov/pubchem/substance/SID11538199> | <http://rdf.ncbi.nlm.nih.gov/pubchem/source/MMDB> |
| <http://rdf.ncbi.nlm.nih.gov/pubchem/substance/SID17137106> | <http://rdf.ncbi.nlm.nih.gov/pubchem/source/SMID> |
| <http://rdf.ncbi.nlm.nih.gov/pubchem/substance/SID836841> | <http://rdf.ncbi.nlm.nih.gov/pubchem/source/MMDB> |
| <http://rdf.ncbi.nlm.nih.gov/pubchem/substance/SID8020778> | <http://rdf.ncbi.nlm.nih.gov/pubchem/source/MMDB> |
| <http://rdf.ncbi.nlm.nih.gov/pubchem/substance/SID48426145> | <http://rdf.ncbi.nlm.nih.gov/pubchem/source/MMDB> |
| <http://rdf.ncbi.nlm.nih.gov/pubchem/substance/SID99289496> | <http://rdf.ncbi.nlm.nih.gov/pubchem/source/MMDB> |
| <http://rdf.ncbi.nlm.nih.gov/pubchem/substance/SID135383529> | <http://rdf.ncbi.nlm.nih.gov/pubchem/source/MMDB> |
| <http://rdf.ncbi.nlm.nih.gov/pubchem/substance/SID136348691> | <http://rdf.ncbi.nlm.nih.gov/pubchem/source/MMDB> |
| <http://rdf.ncbi.nlm.nih.gov/pubchem/substance/SID174861535> | <http://rdf.ncbi.nlm.nih.gov/pubchem/source/MMDB> |
| <http://rdf.ncbi.nlm.nih.gov/pubchem/substance/SID181085729> | <http://rdf.ncbi.nlm.nih.gov/pubchem/source/MMDB> |
| <http://rdf.ncbi.nlm.nih.gov/pubchem/substance/SID181085740> | <http://rdf.ncbi.nlm.nih.gov/pubchem/source/MMDB> |
| <http://rdf.ncbi.nlm.nih.gov/pubchem/substance/SID181085820> | <http://rdf.ncbi.nlm.nih.gov/pubchem/source/MMDB> |
| <http://rdf.ncbi.nlm.nih.gov/pubchem/substance/SID172386498> | <http://rdf.ncbi.nlm.nih.gov/pubchem/source/MMDB> |
| <http://rdf.ncbi.nlm.nih.gov/pubchem/substance/SID7890377> | <http://rdf.ncbi.nlm.nih.gov/pubchem/source/SMID> |

**Table S3.** The federated query result obtained for SID103554720, including the relations, values, units and corresponding ChEMBL assays of bioactivities.

| rel*^a^* | value | unit | label*^b^* |
| --- | --- | --- | --- |
| = | 6 | nM | CHEMBL832902 |
| = | 14 | % | CHEMBL926687 |
| = | 12 | nM | CHEMBL926244 |
| = | 66 | % | CHEMBL927862 |
| = | 175100 | nM | CHEMBL927863 |
| = | 0.9900000095367432 | nM | CHEMBL944784 |
| = | 142 | nM | CHEMBL944785 |
| = | 144 |  | CHEMBL944786 |
| = | -75.19999694824219 | % | CHEMBL944787 |
| = | 3.799999952316284 | nM | CHEMBL944783 |
| = | 30 | % | CHEMBL969502 |
| = | 2.5 | nM | CHEMBL975203 |
| = | 0.2599999904632568 | mg kg-1 | CHEMBL975209 |
| = | 3.759999990463257 |  | CHEMBL975206 |
| = | 6.199999809265137 | nM | CHEMBL975202 |
| = | 78 | nM | CHEMBL975204 |
| = | 1.200000047683716 |  | CHEMBL975207 |
| = | 100 | mg kg-1 | CHEMBL975213 |
| > | 70 | % | CHEMBL969503 |
| = | 31 |  | CHEMBL975205 |
| = | 55 | mg kg-1 | CHEMBL975210 |
| = | 1.950000047683716 | ug L-1 | CHEMBL960527 |
| = | 27 | % | CHEMBL960528 |
| = | 2 |  | CHEMBL975214 |
| = | 100 | mg kg-1 | CHEMBL969490 |
| = | 384 |  | CHEMBL969491 |
| = | 0.2639999985694885 |  | CHEMBL1226551 |
| = | -63 | % | CHEMBL1272354 |
| = | -14 | % | CHEMBL1272355 |
| = | 15 | % | CHEMBL1272356 |
| = | 35 | % | CHEMBL1272357 |
| = | 1.529999971389771 |  | CHEMBL1292029 |
| = | 7079.5 | nM | CHEMBL1613769 |
| = | 112202 | nM | CHEMBL1613776 |
| = | 2500 | nM | CHEMBL1667139 |
| = | 2500 | nM | CHEMBL1667141 |
| = | 2500 | nM | CHEMBL1667144 |
| = | 2500 | nM | CHEMBL1667146 |
| = | 2500 | nM | CHEMBL1667151 |
| = | 2500 | nM | CHEMBL1667152 |
| = | 2500 | nM | CHEMBL1667154 |
| = | 2500 | nM | CHEMBL1667159 |
| = | 9000 | nM | CHEMBL1667161 |
| = | 5900 | nM | CHEMBL1667164 |
| = | 8200 | nM | CHEMBL1667304 |
| = | 10800 | nM | CHEMBL1667305 |
| = | 8000 | nM | CHEMBL1667306 |
| = | 6700 | nM | CHEMBL1667314 |
| = | 6000 | nM | CHEMBL1667315 |
| = | 8600 | nM | CHEMBL1667317 |
| = | 6900 | nM | CHEMBL1667318 |
| = | 7600 | nM | CHEMBL1667320 |
| = | 17000 | nM | CHEMBL1663081 |
| = | 2500 | nM | CHEMBL1667140 |
| = | 2500 | nM | CHEMBL1667143 |
| = | 2500 | nM | CHEMBL1667148 |
| = | 2500 | nM | CHEMBL1667157 |
| = | 2500 | nM | CHEMBL1667160 |
| = | 7100 | nM | CHEMBL1667308 |
| = | 5900 | nM | CHEMBL1667310 |
| = | 7300 | nM | CHEMBL1667312 |
| = | 6900 | nM | CHEMBL1667313 |
| = | 8200 | nM | CHEMBL1667319 |
| = | 25000 | nM | CHEMBL1663080 |
| = | 17000 | nM | CHEMBL1663082 |
| = | 68000 | nM | CHEMBL1663080 |
| = | 39000 | nM | CHEMBL1663081 |
| = | 39000 | nM | CHEMBL1663082 |
| = | 2500 | nM | CHEMBL1667142 |
| = | 2500 | nM | CHEMBL1667145 |
| = | 2500 | nM | CHEMBL1667147 |
| = | 2500 | nM | CHEMBL1667149 |
| = | 2500 | nM | CHEMBL1667150 |
| = | 2500 | nM | CHEMBL1667153 |
| = | 2500 | nM | CHEMBL1667155 |
| = | 2500 | nM | CHEMBL1667156 |
| = | 2500 | nM | CHEMBL1667158 |
| = | 5900 | nM | CHEMBL1667162 |
| = | 5900 | nM | CHEMBL1667163 |
| = | 8700 | nM | CHEMBL1667165 |
| = | 5800 | nM | CHEMBL1667307 |
| = | 2500 | nM | CHEMBL1667309 |
| = | 9700 | nM | CHEMBL1667311 |
| = | 6900 | nM | CHEMBL1667316 |
| = | 9.5 | % | CHEMBL1681272 |
| = | 5100 | nM | CHEMBL1678715 |
| >= | 4 |  | CHEMBL1697798 |
| >= | 4 |  | CHEMBL1697799 |
| >= | 4 |  | CHEMBL1697800 |
| = | 2.5 |  | CHEMBL1697801 |
| >= | 4 |  | CHEMBL1697801 |
| >= | 4 |  | CHEMBL1697802 |
| = | 6.199999809265137 |  | CHEMBL1697802 |
| = | 3 |  | CHEMBL1697797 |
| = | 1.700000047683716 |  | CHEMBL1697798 |
| = | 6.5 |  | CHEMBL1697799 |
| = | 7.300000190734863 |  | CHEMBL1697800 |

*^a^* rel stands for relation between the bioactivity and its value; *^b^* label stands for the ChEMBL assay identifier.
